# Mechanism Underlying the Spatial Pattern Formation of Dominant Tree Species in a Natural Secondary Forest

**DOI:** 10.1371/journal.pone.0152596

**Published:** 2016-03-30

**Authors:** Guodong Jia, Xinxiao Yu, Dengxing Fan, Jianbo Jia

**Affiliations:** Key Lab of Soil and Water Conservation and Desertification Combating, Ministry of Education, College of Soil and Water Conservation, Beijing Forestry University, Haidian District, Beijing, PR China; University of Vigo, SPAIN

## Abstract

Studying the spatial pattern of plant species may provide significant insights into processes and mechanisms that maintain stand stability. To better understand the dynamics of naturally regenerated secondary forests, univariate and bivariate Ripley’s *L(r)* functions were employed to evaluate intra-/interspecific relationships of four dominant tree species (*Populus davidiana*, *Betula platyphylla*, *Larix gmelinii* and *Acer mono*) and to distinguish the underlying mechanism of spatial distribution. The results showed that the distribution of soil, water and nutrients was not fragmented but presented clear gradients. An overall aggregated distribution existed at most distances. No correlation was found between the spatial pattern of soil conditions and that of trees. Both positive and negative intra- and interspecific relationships were found between different DBH classes at various distances. Large trees did not show systematic inhibition of the saplings. By contrast, the inhibition intensified as the height differences increased between the compared pairs. Except for *Larix*, universal inhibition of saplings by upper layer trees occurred among other species, and this reflected the vertical competition for light. Therefore, we believe that competition for light rather than soil nutrients underlies the mechanism driving the formation of stand spatial pattern in the rocky mountainous areas examined.

## Introduction

Secondary forests comprise woody vegetation that recovers naturally after a complete anthropogenic forest clearance [1, 2]. They are valuable remnants as they are vitally important in biodiversity conservation, water conservation, soil erosion control and carbon sequestration. These values of secondary forests have been realized [3] and increasing attention has been paid to the management of such forests. This highlights the importance of studying forest spatial patterns, as ecologically sound forest management approaches should be based on the understanding of the natural patterns and ecological processes underlying these patterns [4]. However, only a few studies have focused on temperate secondary forests [5]. Forest spatial structure yields important clues to understand species interactions with the environment as well as the dynamics of forest communities. In recent years, the development of spatial patterns resulting from intra- and inter-specific associations and environmental factors has been an important topic in ecosystem research [6–9]. It is clear that different determining factors and processes could cause different spatial patterns (aggregated, random, and regular) [10], including facilitation (positive effect) or competition (negative effect), environmental heterogeneity, disturbance and regeneration strategy. An aggregated spatial pattern may be an indication of species having similar ecological requirements [11], facilitation among individuals [12, 13], or dispersal limitations at larger scales [14]. No strong direct influence of any process in shaping spatial interactions has been indicated by random patterns [15]. In productive environments with more available resources where inter- and intra-specific competition would dominate [16], distances between trees tend to be even and the pattern would be altered to become regular [17, 18] Therefore, relationships between spatial patterns and biological processes can help to explain the causal mechanisms that facilitate species coexistence [19, 20]. However, different processes may generate the same spatial pattern; therefore, causal relationships should be carefully stated [21]. For example, previous work showed conclusively that conspecific trees of approximately all of the 1,768 tropical species studied were clumped in spite of the different ecological processes they might experience, which was contrary to what scientists hypothesized in earlier decades [22]. Therefore, it is necessary to distinguish the factors to reveal the mechanism driving the spatial pattern of a stand.

Among the environmental factors plants are simultaneously exposed to, soil resources and light both limit the growth of the plants [23] and thus the spatial formation of the forest. Additional nutrients and water can explain plant productivity [24], and adequate availability of soil resources enables rapid height growth, allowing faster-growing species to grow into a better light environment [25]. Response to changing light levels has been recommended as a potential mechanism for the maintenance of species richness [26]. Competition for light was proved to be even more important than competition for soil nutrients in limiting the growth of species during early succession [27]. In contrast to the size-proportionate nature of soil-related resources acquisition, competition for light is considered to be disproportionate to size. Short individuals receive light disproportionate to their size due to deprivation resulting from their taller neighbors [28, 29]. Given the importance of these light and soil nutrients, their spatial heterogeneity and availability would act as a driving force for the formation of the spatial pattern.

Evaluating the influence of environmental factors on the forest structure has been a persistent theme in forest ecology, and is crucial for forest management practices. However, insufficient studies have been conducted in temperate secondary forests. Addressing the driving mechanism of stand structure, the primary objective of this work was to explore the possible influences of soil conditions and light on the spatial pattern of trees at different life stages by studying the intra- and inter-specific relationship of the dominant species of different height and diameter classes. Specifically, we analyzed (1) whether there was a corresponding relationship between soil and trees by analyzing the spatial pattern of soil moisture, pH and nutrients and that of the four dominant species, namely *Populus davidiana*, *Betula platyphylla*, *Larix gmelinii* and *Acer mono*; and (2) the effect of light-blocking by adult trees on saplings/young trees in the formation of forest spatial patterns, which would be an interesting and novel contribution to the understanding of the relationship between the environmental factors. We hypothesized that saplings would not cluster near taller adults, regardless of species, and then determined whether this relationship would hold when accounting for microhabitat soil characteristics. The study will be helpful in determining the mechanisms important in structuring secondary forest communities because such patterns can reflect underlying processes, such as establishment, growth and competition. This should ultimately allow us to make better informed decisions about the approaches that should be adopted during silvicultural treatments.

## Materials and Methods

### Study Area and Vegetation Survey

The study site is located at a state-owned forest station in the Mulan paddock (41°35′-42°40′N, 116°32′-117°14′E, 750-1998m a.s.l.) of Weichang county, Hebei province, approximately 360 km northeast of Beijing. The station obtains the permission from national State Forestry Administration of China. And, the study was allowed by the management office on this site and the field studies did not involve endangered or protected species. It lies in the transition belt from the cold to warm temperate zone and is under the control of the continental monsoon climate. The average annual temperature is 4 °C and the average annual precipitation ranges from 380-560mm, occurring mainly from July to September. The region is dominated by temperate deciduous forests. There was an intensive harvest campaign targeting medium and large trees in this region between the 1940s and 1950s. Eighty five percent of the farm is naturally regenerated secondary forests, covering an area of 1485 ha.

A 200 m×200 m plot was established in July 2014 in the forest, divided into sixteen 50 m×50 m sub-plots. In order to facilitate data collection, each sub-plot was further divided into 10 m×10 m (for the vegetation survey, Table 1) and then 5 m×5 m quadrats (for obtaining accurate coordinates of individual trees). Each tree greater than or equal to 1cm DBH (diameter at breast height, at 1.3 m height) was assigned a pair of x and y coordinates in the plot, and the corresponding species, DBH and crown diameter data were recorded.

**Table 1:** Characteristics of the four dominant species in the secondary forest within the survey area covering 4ha.

Species	Number of trees	Height(m)		DBH(cm)		Canopy area (m^2^)	
		Max./Min.	Average	Max./Min.	Average	Max./Min.	Average
***Populus davidiana***	1302	23/2.00	12.28	47.4/2	16.15	127.61/1.04	13.21
***Betula platyphylla***	927	25.8/1.80	12.53	47.5/1.5	16.66	182/1.04	13.35
***Larix gmelinii***	290	25/2.20	14.20	54.7/2	23.72	33.17/0.63	8.81
***Acer mono***	179	15.6/2.50	6.76	38/2.3	8.41	63.58/1.03	13.66

### Spatial Distribution of Soil Water and Nutrients

In order to determine the spatial distribution of soil water and nutrients, values obtained from different depths were averaged to represent the whole profile. Given the rocky and sandy texture of the soil, the soil water content was only measured to a depth of between 40 and 60cm. For this purpose, portable TDRs (Field Scout TDR200, Spectrum^®^, US) were deployed in the center of each 10 m×10 m quadrat in the subplots, i.e., the same scale as the vegetation survey plot. The sampling was conducted during July and there was no rain during the previous three days. All sampling was finished within the same day to minimize the differences between quadrats. The soil samples were collected at different depths (0–20 cm、20–40 cm、40–60 cm), using a soil drill, to determine the soil nutrients, including pH, soil organic matter (SOM), available phosphorus, available potassium, total nitrogen, total phosphorus and total potassium. Due to the limit in manpower, the sampling unit of soil nutrient was extended to 20 m×20 m quadrat. And in some quadrats, more than one sample was taken where there were lots of rocks in the drilled samples. The samples were sealed in the plastic bags and transported to the laboratory for nutrient determination. The SOM physical fractionation was exothermically oxidized using a K_2_Cr_2_O_7_ -H_2_SO_4_ solution and titrated with Fe_2_SO_4_ [30] Soil pH was determined with a glass electrode using a soil:water ratio of 1:2.5 [31]. Soil available P was extracted by sodium bicarbonate and determined using the molybdenum blue method [32]. Soil available K was extracted by ammonium acetate and determined by flame photometry [33], and total N was determined by Kjeldahl digestion [30]. Interpolation analysis was conducted in ArcGIS 10.2 using the soil water/nutrient data (Table 2). We used Original Kriging as the interpolation approach because it can fully use the information from the samples and provide comprehensive considerations of the spatial location, size, and distance interval.

**Table 2:** Descriptive statistics of soil traits from samples across the 4-ha plot area.

Soil Traits (unit)	Max.	Min.	Average	Median	S.D.	C.V.
**Water Content (%)**	38.2	0.8	22.57	24.4	7.19	0.32
**pH**	6.90	5.06	6.03	6.02	0.31	0.05
**SOM (g/kg)**	129.22	2.74	29.09	25.02	20.52	0.71
**Available Phosphorus (mg/kg)**	59.46	2.17	9.57	7.82	7.30	0.76
**Available Potassium (mg/kg)**	367.35	51.02	139.78	126.53	62.69	0.45
**Total Phosphorus (g/kg)**	0.95	0.06	0.38	0.34	0.19	0.51
**Total Potassium (g/kg)**	26.82	10.57	17.60	17.38	3.27	0.19
**Total Nitrogen (g/kg)**	4.17	0.42	1.53	1.45	0.68	0.44

Spearman 's rank correlation coefficient was used to test whether the occurrence of trees of certain DBH/ height classes was related to soil nutrients [34], Indices representing soil conditions were correlated with the number of individuals of different DBH/height classes.

### Analysis of Stand Spatial Pattern and Association

Because ecological processes are unlikely to be mutually exclusive, spatial patterns should be analyzed at multiple spatial scales to enhance our understanding of species coexistence and community structure [21]. Ripley’s *K(r)* function [35] analyzes spatial patterns and species associations at different scales and has been extensively applied in community spatial pattern analysis [36–39].

Univariate analysis of Ripley’s *K(r)* function, which characterizes spatial patterns at a range of distances, was performed to examine the spatial pattern of the same tree species or of the same DBH/height class

$$K(r)=\frac{A}{n^{2}}\sum_{i=1}^{n}\sum_{j=1}^{n}\frac{I_{r}(u_{ij})}{w_{ij}}$$ (1)

Where *r* is the distance between plants, *A* the area of the plot, *n* is the number of individuals within the plot, *u*_*ij*_ is the distance between individuals *I* and *j*; and *w*_*ij*_ is defined as the inverse of the proportion of is a weighting factor correcting for edge effects. The weighting factor is the proportion of the circumference of the circle centered on point i and passing through point j, which is inside the bounds of the study area. *K(r)* was linearized by square root transformation *L(r)* to stabilize the variance and simplify the interpretation of *K(r)*:

$$L(r)=\sqrt{\frac{K(r)}{\pi }}-r$$ (2)

where *r* is the distance between trees. For a univariate point pattern, a simulation envelope of *L(r)* was developed through Monte Carlo simulations based on a homogenous Poisson process [40]. Plant individuals were aggregated at distances *r* when *L(r)* exceeded the upper limit of the interval. Individuals were uniform at distances *r* when *L(r)* was below the lower limit of the confidence interval; otherwise, individuals were randomly distributed.

If the spatial pattern of the population was aggregated, the value of *L(r)* that deviated from the upper limit of the confidence interval was defined as the degree of aggregation and the corresponding distance was the aggregation distance [41]. The aggregation dimension was defined as the area of a circle with the radius of the corresponding aggregation distance.

Bivariate analysis Bivariate Ripley’s *K(r)* functions were used to evaluate the spatial associations between two species or between trees of different DBH/height classes. The functions were defined as:

$$K_{12}(r)=\frac{A}{n_{1}n_{2}}\sum_{i=1}^{n_{1}}\sum_{j=1}^{n_{2}}\frac{I_{r}(u_{ij})}{w_{ij}}$$ (3)

where *1*,*2* represent the two species and *n*_*1*_,*n*_*2*_ are the number of individuals of each species. The other symbols are the same as in (1). *L*_*1*,*2*_ *(r)* is a substitute for *K*_*1*,*2*_*(r);* the functions are the same as in (2).

For the bivariate case, when values of *L*_*1*,*2*_*(r)* fall in the simulation envelope of *L(r)*, the two groups are considered to be independent (no interaction). If *L*_*1*,*2*_*(r)* is greater than the upper limit of the simulation envelope of *L(r)*, the two groups are positively associated, meaning there is an attraction between the two groups. If *L*_*1*,*2*_*(r)* falls below the lower limit of the confidence interval, the association between the two groups is negative, indicating a repulsion between the two groups of trees.

The analyses were performed using the software package Programita [21]. We adopted CSR (Complete Spatial Randomness) for univariate analysis because environmental heterogeneity was limited [42]. We hypothesized that the taller/larger trees would influence the distribution of the shorter/smaller trees but not vice versa. Therefore, the null model was set to be pattern 1 fixed-pattern 2 random for the analysis of the inter-DBH/height class. Calculation of the 95% simulation intervals was based on the 5th-lowest and 5th-highest values of 99 Monte Carlo simulations.

## Results

### Forest Stand Structure

**Fig 1:**
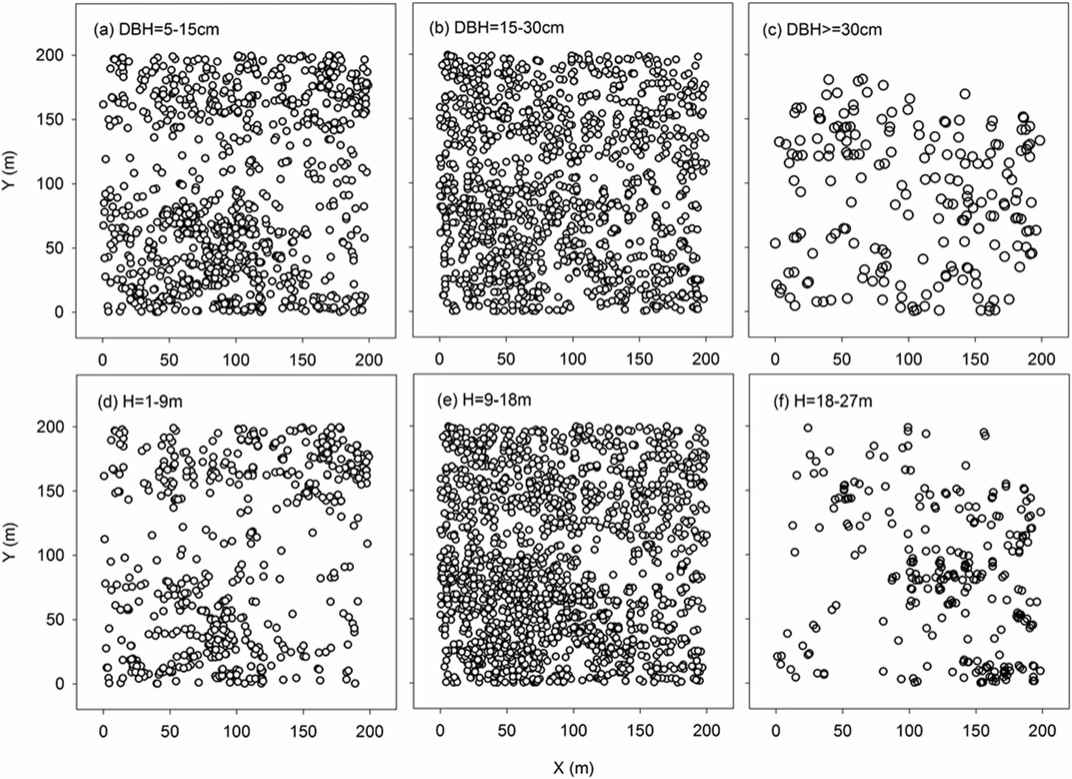
Spatial distributions of trees of different DBH classes (small (a), medium (b), large (c)) and height classes (sapling (d), medium (e), tall (f)).

**Fig 2:**
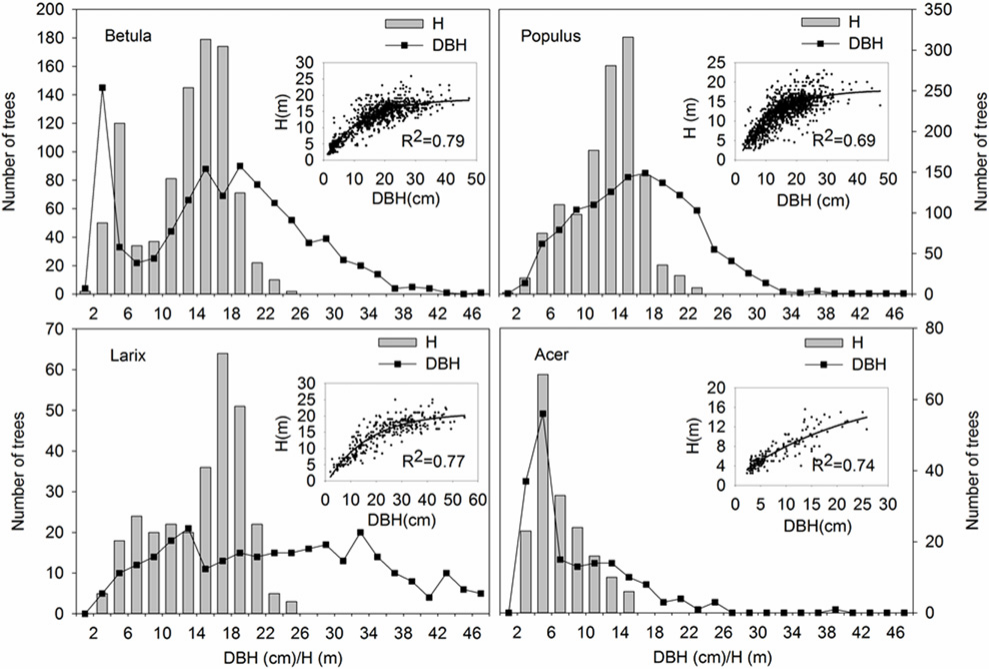
Statistics of trees in each DBH and height interval and the corresponding relationship between DBH and height (embedded) for the four dominant species.

The stand was forested predominantly with *Populus davidiana*, *Betula platyphylla*, *Larix gmelinii* and *Acer mono*. A non-uniform distribution was observed for the dominant species at different life stages (Fig 1). On an average level, *Larix* was taller and larger than the other three species (Table 1); the quantity composition also showed that the height and DBH peaks of *Larix* were biased towards the upper end (Fig 2). *Populus* and *Betula* were similar in average height and DBH (Table 1), but in contrast to the single DBH and height distribution peak of *Populus*, a double peak was observed for *Betula* with one at 2–6 cm and the other at 14–18 cm (Fig 2). The *Acer* population was mainly composed of small individuals (Table 1 and Fig 2). For all species, the height increased exponentially with DBH (Fig 2 embedded). In contrast to the other three species, however, the height of *Acer* had not reached the maximum.

### Spatial Pattern of Soil Water and Nutrients

**Fig 3:**
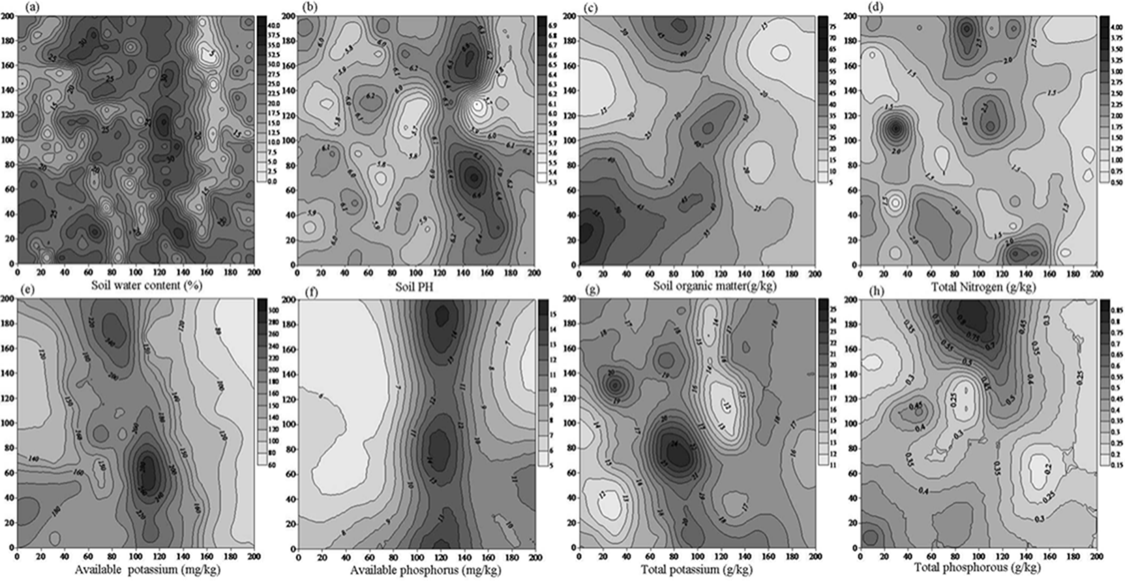
Spatial distribution of soil physical and chemical indices.

Interpolation results showed that the soil water content was distributed relatively evenly with limited fragments below 5% or above 30% (Fig 3A). The soil pH was generally below 6.3 across the plot (Fig 3B), and the soil organic matter fell within the range of 60-95g/kg (Fig 3C). A high content of available potassium (>250 mg/kg) was distributed across the middle part of the plot and decreased horizontally towards the sides (Fig 3E). Total potassium was 16–19 g/kg in most areas of the plot with a small area above 25 g/kg or below 13 g/kg (Fig 3G). The available phosphorous in most areas was below 9 mg/kg. A high content of available phosphorus (>14 mg/kg) occurred in the middle part of the plot (Fig 3F). The highest level (>0.8g/kg) of total phosphorus was measured in the upper part of the plot, and fragments of low total phosphorus content were distributed sporadically (Fig 3H). In contrast to the spatial continuity of the other soil properties, the distribution of total nitrogen was more fragmented and exhibited a complex spatial pattern (Fig 3D). No relationship was found between the spatial pattern of the soil nutrients and that of trees of different sizes and height (Table 3).

**Table 3:** Spearman’s rank correlation coefficients (P level) of tree distribution and the spatial pattern of soil nutrients, i.e. correlation between the number of individuals of different DBH/height classes and the soil condition indices. The correlation is significant if P<0.05.

Soil Condition indices	DBH class			Height class		
	5-15cm	15-30cm	> = 30cm	1-9m	9-18m	18-27m
**Water Content**	0.609(0.061)	0.096(0.062)	0.178(0.068)	0.527(0.057)	0.860(0.065)	0.109(0.095)
**pH**	0.825(0.076)	0.864(0.053)	0.347(0.056)	0.406(0.081)	0.720(0.077)	0.612(0.186)
**SOM**	0.137(0.068)	0.240(0.068)	0.890(0.059)	0.358(0.070)	0.115(0.051)	0.184(0.053)
**Available Phosphorus**	0.334(0.057)	0.383(0.058)	0.174(0.075)	0.536(0.059)	0.115(0.073)	0.874(0.063)
**Available Potassium**	0.839(0.052)	0.371(0.085)	0.513(0.097)	0.921(0.080)	0.833(0.082)	0.195(0.083)
**Total Phosphorus**	0.515(0.089)	0.756(0.059)	0.869(0.089)	0.921(0.068)	0.768(0.084)	0.281(0.067)
**Total Potassium**	0.642(0.062)	0.033(0.090)	0.067(0.068)	0.321(0.063)	0.867(0.090)	0.455(0.069)
**Total Nitrogen**	0.729(0.054)	0.523(0.053)	0.980(0.051)	0.919(0.069)	0.428(0.058)	0.078(0.193)

### Intra- and Inter-species Relationship of Dominant Species

**Fig 4:**
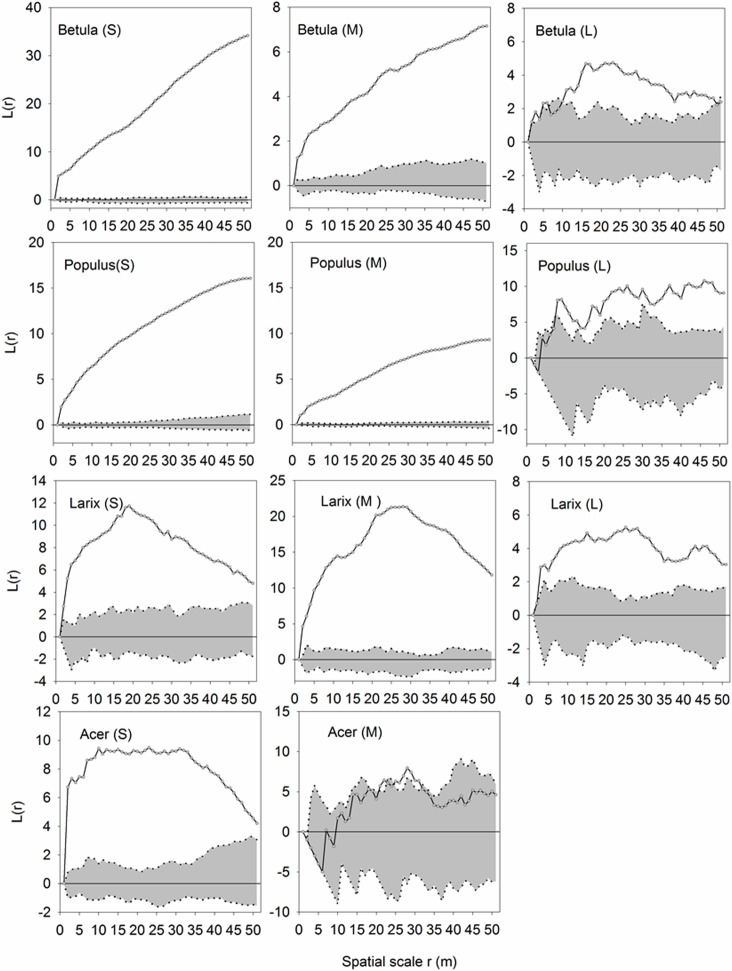
Univariate analyses of trees of different DBH classes. Univariate functions *L*(r) (dotted lines) are shown with the simulation intervals (shadow areas). L: large (DBH = 1-15cm); M: middle (DBH = 15-30cm); S: small (DBH> = 30cm).

**Fig 5:**
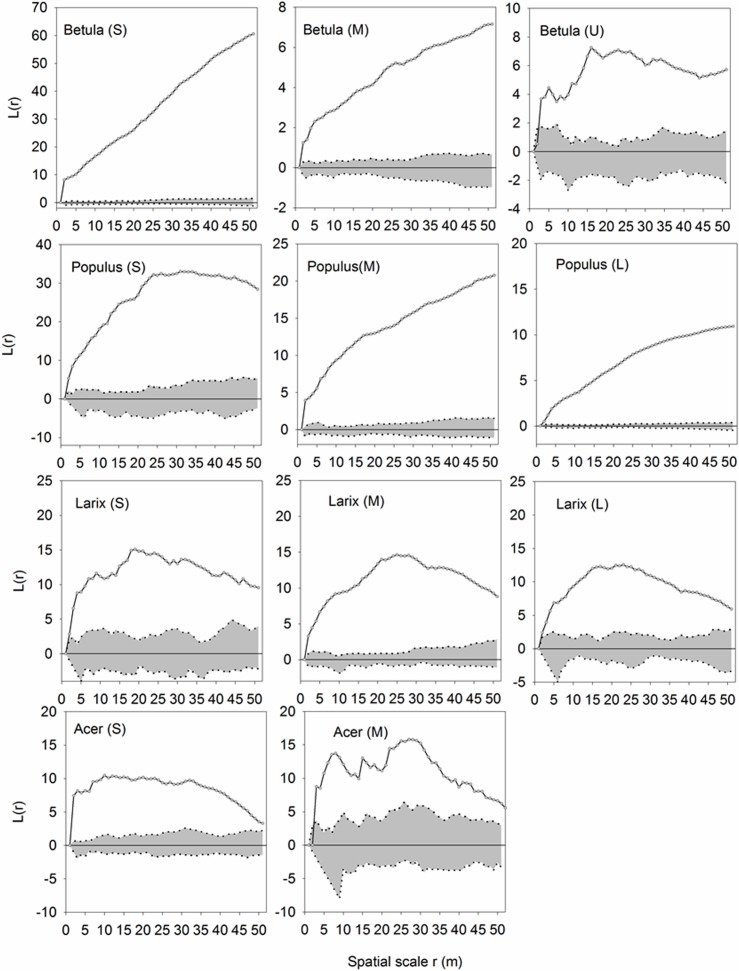
Univariate analyses of trees of different heights. Univariate functions *L*(r) (dotted lines) are shown with the simulation intervals (shadow areas). U: upper layer (H = 18-27m), M: middle layer (H = 9-18m); S: sapling (H = 1-9m).

Regardless of species, trees of different sizes were strongly aggregated at almost all distances (Fig 4). However, the aggregation decreased as the tree size increased. In the case of *Acer*, the aggregated pattern of small trees shifted to random when the medium trees were examined. Despite the significant correlation between size and height, this transition in spatial pattern was observed only among different height classes of *Betula* and *Populus* (Fig 5).

**Fig 6:**
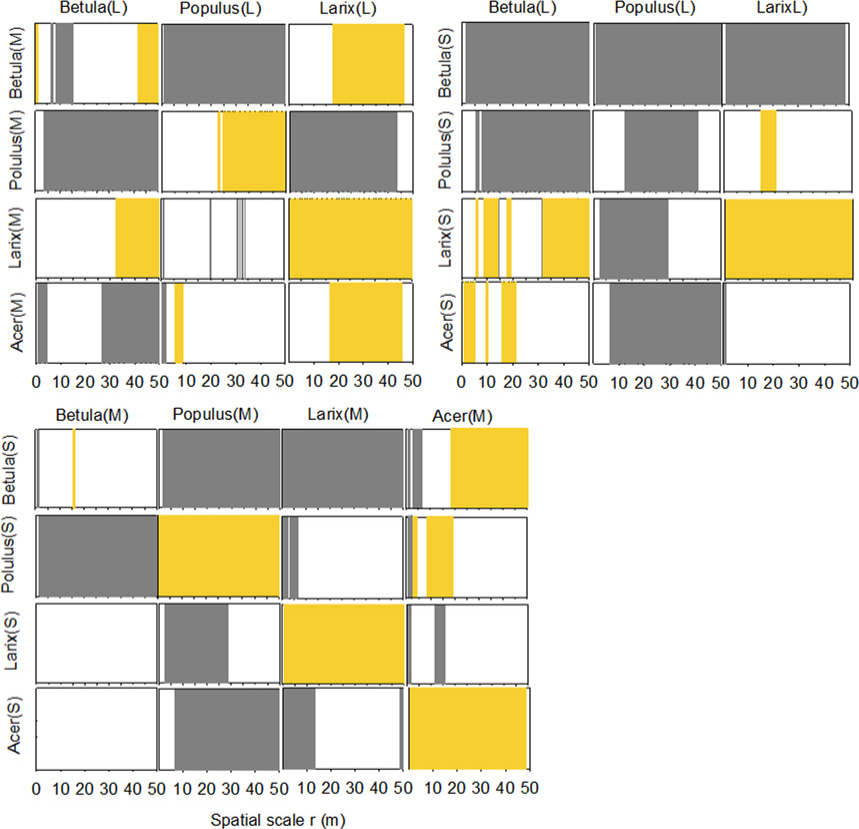
Bivariate analyses exploring the association of intra- and interspecies relationship between trees of different DBH ranks. The classification of DBH classes was the same as in Fig 4. L: large; M: middle; S: small. Grey bars represent for repulsion; yellow bars represent attraction, and the voids represent for independence.

The intraspecific relationship between trees of different DBH classes was case-dependent (Fig 6). *Betula* trees of the middle DBH class were drawn to and then repelled by the trees of the large DBH class at small scales. They became independent from 12m to 40m and attracted across a large scale (>42m). In the *Populus* population, a positive interaction was found between individuals with middle and large DBH classes at distances over 22m. By contrast, individuals of the small DBH classes were depressed by those of the middle and large DBH classes. In the *Larix* population, intra-species spatial attraction was found between trees of different DBH classes across all distances. The same pattern was also found for *Acer*.

Bivariate analysis revealed that the large trees of *Populus* and *Betula* were mutually repulsed by the middle and small sized individuals of each other. Under most circumstances, *Larix* was either independent of or attracted to the large and middle trees of other species, and the inhibition was posed on this species by *Populus* and middle-DBH *Acer* trees at a distance <30m. At most distances, *Acer* had no negative influences on the small trees.

**Fig 7:**
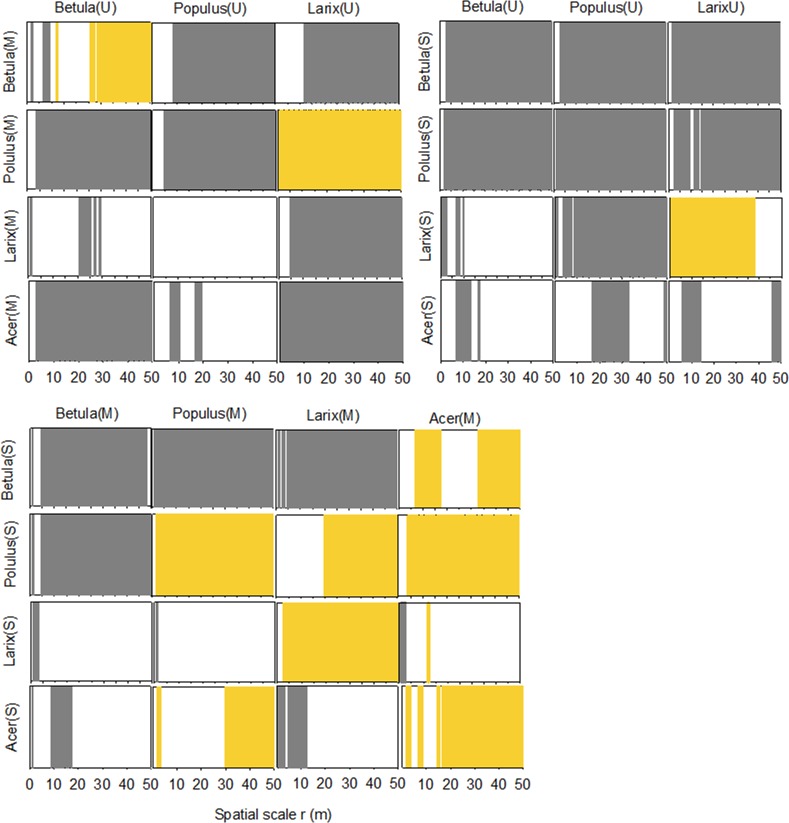
Bivariate analyses exploring the association of intra- and interspecies relationships between trees of different height ranks. The classification of height classes was the same as in Fig 4. U: upper layer, M: middle layer; S: sapling. Grey bars: repulsion; yellow bars: attraction; void: independence.

Except for *Betula* (U)-(M) and *Larix* (U)-(M), upper layer trees exerted inhibition on middle layer trees at most distances (Fig 7). The height-related inhibition was more significant with respect to the effect of upper layer trees on saplings. Except for *Larix*, a negative relationship was presented at different scales. In the comparison between middle layer trees and saplings, inhibition from taller trees weakened and shifted to facilitation in many pairs. Except for *Betula*, saplings of the other species presented positive association with their conspecific middle layer adults from small to large scales (5–30m).

## Discussion

Except *Larix*, the other three species are deciduous broadleaf species. All four species are heliophytes and seed dispersal is aided by wind, which means that the dispersal limitation of the four species is the similar. Specifically, *Populus davidiana* flowers in early spring, before the leaves are produced; numerous minute light brown seeds surrounded by long, soft, white hairs that aid broadcasting by wind [43]. *Betula platyphylla* is a pioneer tree species in waste land and the winged-nuts can be easily dispersed by wind [44]. *Larix gmelinii* is a deciduous coniferous species widely spread across the northern temperate forest. The cones turn brown and open to release the seeds when mature, but the cones usually remain on the tree and winged-seeds are spread by wind [45]. *Acer mono* is a deciduous broad-leaf species. Their samara fruits are oval shaped [46].

### Intra- and Interspecific Relationships

Study of the spatial pattern of individuals within and among tree species is important for the determination of interactions between trees. Therefore, spatial patterns can be helpful in understanding the mechanisms that maintain tree species coexistence. Positive interactions have been reported in harsh environments, e.g., alpine [47] and mountainous forest communities [48–50]. In our study, an overall positive spatial relationship was found between trees of the same DBH or height class regardless of species (Fig 4). The spatial aggregation could be attributed to several major mechanisms and processes [10] that include habitat heterogeneity [51] and dispersal limitation [52].We found a gradual shifting of aggregated distributions to random as the tree size or height increased, such as in the cases of *Betula* and *Populus*. This pattern likely emerged because trees with smaller DBH and lower height classes require fewer resources, and thus afforded sharing of limited resources by coexisting counterparts. Random or regular distributions were observed in *Acer* with medium DBH classes. Similar results were obtained by previous research [53]. This can be attributed to the fact that compared to juveniles, adult trees with a large size or in the upper layer require more resources, such as light, water, and nutrients, to sustain them [54, 55]. The limited available resources cannot meet the demand of all trees; therefore, trigger “self-thinning” is triggered, which causes the distribution to become increasingly regular [9, 17, 56–58].

Interspecific spatial association between different height classes was dominated by negative association at most distances. Mutual repulsion was also more likely to emerge in the pairing between of large vs. medium or small sized trees, demonstrating that the effect of inhibition on juveniles increased as the trees matured. The negative association was attributed to taller trees which obtained more light resources than seedlings; hence competition was the main intraspecific dynamic of the trees studied (Figs 6 and 7). Bivariate Ripley’s *L(r)* analysis indicated that *Populus* and *Betula* were negatively associated with other species at most spatial scales. This could be attributed to the fact that they both are light-demanding species sharing the same space; hence, competition for resources was intense.

Sapling distribution was found to be either inhibited by or independent of that of adult trees, especially those in large DBH or height classes. The wind-dispersed samara fruit should be broadcast away from the conspecific trees. However, for *Larix*, the small trees tended to aggregate around the middle and large DBH/height trees. Moreover, this result contradicted the Janzen-Connell hypothesis [59], which proposed that distance from the parent trees facilitates the survival and establishment of the seedlings. We believe the microhabitat near the parent trees facilitates seedling establishment; otherwise, they would have been found away from the adults. A similar pattern was reported in the temperate upland hardwoods where conspecific trees were aggregated in most cases except for some degree of overdispersion [60]. In our site, the conical canopy of *Larix* enables more sunshine to reach the understorey, and thus diminishes the negative impact of light shortage with respect to the conspecific seedlings. The light-manipulation with respect to the distribution of the *Larix* seedlings was further evidenced by their relationship with other species (Fig 7). Therefore, compared to the other trees, the aggregation of seedlings around the parent trees can be regarded as evidence that conspecific adult trees created a light-facilitating environment for seedlings. The other three broad-leaved species had expanded canopies (Table 1) and intercepted sunshine. The seedlings were inhibited by or independent of the other species in the upper and middle layers. Under a denser canopy layer dominated by *Populus* and *Betula*, seedlings were inhibited due to lower light levels than in small to medium sized gaps [61]. A similar phenomenon was observed in beech and fir forests. A negative interaction was found between overstorey and understorey beech trees at short distances but not between overstorey fir and understorey beech. The spatial segregation between juvenile and mature beech was attributed to the dependence of the juvenile trees on light availability [62]. In our study, all four species broadcasts their wind-dispersed winged seed pods to rapidly re-establish as a pioneer tree species on the bare soil exposed to full sunlight in the early stages of succession. The ability of these species to grow rapidly under full sunlight carries with it an inability to regenerate under a continuous canopy cover. Therefore, we believe the light conditions near the parent trees impeded seedling establishment and survival, congruent with the Janzen-Connell hypothesis [59].

### Driving Mechanism for Spatial Patterns and Implications

Our results indicated that the forest system studied in this work was still young and at a growing stage. All the studied species had numerous juveniles. Peaks in the small DBH and height classes of *Betula* and *Acer* indicated that the species began self-renewal. A similar pattern was reported in 167 stands of lodgepole pine in south central British Columbia [63]. Our results showed that light rather than soil factors was the major determinant of spatial pattern formation in this secondary forest. The distribution of soil water and nutrients demonstrated clear gradients (Fig 3). However, no resemblance was found in the spatial pattern of the trees with different DBH or height classes (Table 3). This indicated that soil conditions did not constitute limitations for trees to compete. This was also reflected by the systematic absence of an inhibitory effect of the adult trees on the juveniles (Fig 6). The relationships between tree distribution and subtle differences in soil properties within a given landscape unit have received much less attention [64]. Similar to our finding, a study in a neotropical forest demonstrated that soil type was not significantly related to the diameter growth [65]. In fact, among the limited research in this area, most studies were focused on the relationship between soil properties and species instead of diameter or height. For example, a study on *Nothofagus* forests in Tierra del Fuego habitats of different soil fertility suited different species [66]. But unlike our site, waterlogging conditions existed in one of their studying sites, and thus appeared to have large effects on the cycling of N. By contrast, a review demonstrated that only 3 out of 18 studies indicated a correlation between soil chemical properties and species composition [67]. Such contradictions among the studies and between theirs and ours can be attributed to the failure to measure available nutrients that can be used by the plants. It was proposed that trees vary in their efficiency of using nutrients [66]. In contrast to soil, the influence of light was obvious. Inhibition was found in almost every case of paired analysis among different height classes, especially between upper layer trees and saplings (Fig 7), indicating a light-blocking effect of adult trees on saplings. Therefore, our hypothesis was validated. This meant that light availability was the major environmental factor that drove the formation of the stand spatial pattern. As a vertically reallocated resource, light was intercepted by the canopy layers and thus constituted a limitation to the saplings. Based on the absence of an influence of soil nutrients on the distribution of trees of different sizes/heights, we believe that light availability rather than soil nutrients status is the major determinant for the formation of spatial pattern. Similarly, a mechanism-incorporated model indicated that a large number of trees per hectare suggests competition for light overwhelmed soil nutrient availability in determining the establishment of seedling development [68].

Based on the dynamics that formed the spatial pattern, for the management of the forest in this study, an increase in the understory light conditions, such as the removal of the competitors from upper layers or canopy trimming, would promote stand regeneration and maintain a diverse plant species mixture. This would lower the risk of competitive exclusion of shade-intolerant seedlings by the low height-growth rate species due to light interception by their strong competitors [69].

## Conclusion

We studied and analyzed the size structure, spatial pattern and driving mechanism of the spatial pattern of a secondary forest to generate practical guidelines for future reference of the sustainable management of similar forest ecosystems. The distribution of soil water and nutrients was not severely fragmented, but a clear gradient was found across the study plot. The stand was well developed and presented an overall aggregation at multiple scales. No systematic inhibitive influence was found by trees of large size over small ones, indicating limited influence from competition for soil resources in forming the stand spatial pattern. By contrast, prominent inhibition of small trees caused by tall trees was presented. Combined with the absence of correlation between stand spatial pattern and the distribution of soil water and nutrients, it is reasonable to arrive at the conclusion that competition for light availability constitutes the major determinant of the formation of the stand spatial pattern. Measures that alleviate light competition should facilitate the stability of species composition and the maintenance of species diversity.

## Supporting Information

S1 File Data used for analyze the spatial pattern of the four dominant species and that of the soil water/nutrients. This file contains two sheets: 1. Stand survey that demonstrates the coordinates, DBH, tree height and canopy area; 2. Soil water and nutrients.
(XLSX)
